# Human Transcriptomic Response to the VSV-Vectored Ebola Vaccine

**DOI:** 10.3390/vaccines9020067

**Published:** 2021-01-20

**Authors:** Francesco Santoro, Alessia Donato, Simone Lucchesi, Sara Sorgi, Alice Gerlini, Marielle C. Haks, Tom H. M. Ottenhoff, Patricia Gonzalez-Dias, VSV-EBOVAC Consortium, VSV-EBOPLUS Consortium, Helder I. Nakaya, Angela Huttner, Claire-Anne Siegrist, Donata Medaglini, Gianni Pozzi

**Affiliations:** 1 Laboratory of Molecular Microbiology and Biotechnology (LAMMB), Department of Medical Biotechnologies, University of Siena, 53100 Siena, Italy; alessia.donato@student.unisi.it (A.D.); lucchesi5@student.unisi.it (S.L.); sara.sorgi@student.unisi.it (S.S.); donata.medaglini@unisi.it (D.M.); 2 Microbiotec srl, 53100 Siena, Italy; alice.gerlini@microbiotec.eu; 3 Department of Infectious Diseases, Leiden University Medical Center, 2333 Leiden, The Netherlands; M.C.Haks@lumc.nl (M.C.H.); t.h.m.ottenhoff@lumc.nl (T.H.M.O.); 4 Department of Clinical and Toxicological Analyses, School of Pharmaceutical Sciences, University of São Paulo, São Paulo 05508-020, Brazil; patricia.gonzalez@usp.br (P.G.-D.); hnakaya@usp.br (H.I.N.); 5 Scientific Platform Pasteur-USP, São Paulo 05508-020, Brazil; 6 Department of Pathology and Immunology, Faculty of Medicine, University of Geneva, 1211 Geneva, Switzerland; angela.huttner@hcuge.ch (A.H.); claire-anne.siegrist@unige.ch (C.-A.S.); 7 Infectious Diseases Service, Geneva University Hospitals, 1205 Geneva, Switzerland; 8 World Health Organization Collaborating Centre for Vaccine Immunology, Faculty of Medicine, University of Geneva, 1200 Geneva, Switzerland

**Keywords:** Ebolavirus Disease, transcriptomics, recombinant VSV, VSV-ZEBOV, vaccine, live viral vector

## Abstract

Ebolavirus Disease (EVD) is a severe haemorrhagic fever that occurs in epidemic outbreaks, with a high fatality rate and no specific therapies available. rVSVΔG-ZEBOV-GP (Ervebo^®^), a live-attenuated recombinant vesicular stomatitis virus vector expressing the glycoprotein G of Zaire Ebolavirus, is the first vaccine approved for prevention of EVD. Both innate and adaptive responses are deemed to be involved in vaccine-induced protection, yet the mechanisms are not fully elucidated. A global transcriptomic approach was used to profile the blood host-response in 51 healthy volunteers enrolled in a phase 1/2 clinical trial. Signatures of the host responses were investigated assessing the enrichment in differentially expressed genes (DEGs) of specific “blood transcription modules” (BTM). Comparison of gene-expression levels showed that vaccination produces a peak of 5469 DEGs at day one, representing 38.6% of the expressed genes. Out of 346 BTMs, 144 were significantly affected by vaccination. Innate immunity pathways were induced from day 1 to day 14. At days 2 and 3, neutrophil modules were downregulated and complement-related modules upregulated. T-cell and cell-cycle associated modules were upregulated at days 7 and 14, while at day 28, no modules remained activated. At day 14, a direct correlation was observed between ZEBOV glycoprotein-specific antibody titres and activation of seven BTMs, including two related to B-cell activation and B cell receptor signalling. Transcriptomic analysis identified an rVSVΔG-ZEBOV-GP-induced signature and demonstrated a direct correlation of blood transcriptomic changes with ZEBOV glycoprotein-specific antibody titres.

## 1. Introduction

Ebolavirus Disease (EVD) is a severe haemorrhagic fever that affects both humans and non-human primates and occurs in epidemic outbreaks. The average EVD fatality rate is around 50%, ranging from 25% to 90% in different outbreaks [1], and no specific therapies are currently licensed. During the 2014 EVD outbreak in West Africa, over 28,000 cases and 11,000 deaths have been reported [2,3], while in the 2018–2020 outbreak in the Democratic Republic of Congo (DRC), 3323 confirmed cases and 2299 deaths have occurred [4].

Ervebo^®^ (rVSVΔG-ZEBOV-GP) is the first vaccine approved from both the European Medicines Agency (EMA) [5] and the Food and Drug Administration (FDA) [6] for the prevention of EVD in individuals 18 years of age and older. In addition, the vaccine was as well pre-qualified by the World Health Organization and approved in in the DRC, Burundi, Ghana, and Zambia as of writing.

rVSVΔG-ZEBOV-GP is a recombinant vaccine based on the Vesicular Stomatitis Virus (VSV) from which the original VSV glycoprotein encoding gene was deleted and replaced with the glycoprotein (GP) encoding gene from the Ebolavirus Zaire strain (ZEBOV) [7]. This chimeric rVSVΔG-ZEBOV-GP vaccine therefore expresses on its surface the ZEBOV GP, which directs the viral tropism and influences its interaction with the immune system cells, while conserving the replication apparatus of VSV.

This vaccine has been shown to confer 100% protection in non-human primates [8], even at very low doses [9], and to be effective after injection of a single intramuscular dose in humans [10]. Vaccine efficacy was demonstrated in clinical studies conducted on 15,399 adults in North America [11,12,13], Europe [14], and Africa [15,16]. The vaccine was shown to be safe, even if occasionally associated with transient reactogenicity [14]. It has now been used in over 290,000 subjects, mainly enrolled under Expanded Access ring vaccination protocols conducted during 2018 DRC outbreaks [10,11,17,18]. rVSV-ZEBOV was also used for post-exposure prophylaxis in 26 subjects, where it was well tolerated and immunogenic [19]. Long-term monitoring of rVSVΔG-ZEBOV-GP vaccines revealed that antibody responses to a single-dose were sustained for at least 2 years in both European and African vaccines immunized with different vaccine doses [20].

Despite its demonstrated efficacy, the precise mechanisms of inducing protective immunity remain largely unclear. Anti-GP antibodies most probably play a key role in the vaccine’s high protective efficacy, but vaccine induced correlates of protection have not been fully understood [21,22,23]. Animal challenge experiments and data from ring vaccination trials indicate that protection from EVD occurs early after immunization, suggesting a critical role of innate immunity in the response to rVSVΔG-ZEBOV-GP. An early innate signature including six chemokines and cytokines was found to correlate with both immunogenicity and reactogenicity of rVSVΔG-ZEBOV-GP in European as well as African vaccines [24]. Chemokines and cytokines were related to monocytes, suggesting that monocyte recruitment and activation are essential in the response to rVSVΔG-ZEBOV-GP. A rapid and dose-dependent NK cell modulation was also shown to be elicited by rVSVΔG-ZEBOV-GP [25].

In-depth studies are needed to fully elucidate the role of innate immune responses in the protection conferred by the rVSVΔG-ZEBOV-GP vaccine and transcriptomic analysis can offer a powerful tool to decipher the innate immune pathways involved.

Blood RNA sequencing enables analyses to profile the host response, to identify differentially expressed genes (DEGs) and correlate them to specific immune modules [26]. Using a systems biology approach, high-dimensional RNA-expression data can be integrated with clinical and immunologic phenotypes to identify transcriptional signatures of immunogenicity and reactogenicity, and to elucidate possible vaccine associated mechanisms of immune response [26,27]. This approach has been employed to dissect the mechanism of action of different vaccines in clinical trials [28,29] and in pre-clinical models [30,31,32].

To investigate the features of the early protection elicited by the rVSVΔG-ZEBOV-GP vaccine, we used a global transcriptomic approach to profile the host response. The blood transcriptomic response to high dose vaccination (10^7^ and 5 × 10^7^ pfu) with rVSVΔG-ZEBOV-GP was analysed in 51 volunteers of the phase 1/2 randomised controlled trial (NCT02287480) conducted in Geneva and transcriptomic data were integrated with clinical and immunological data.

## 2. Materials and Methods

### 2.1. Study Design

In the double-blind, phase 1/2, randomised, controlled trial (NCT02287480, started in November 2014) conducted in Geneva, Switzerland, the immunogenicity and safety of rVSVΔG-ZEBOV-GP vaccine were tested in 115, non-pregnant, healthy adults aged 18–65 years [14,16]. Participants received either vaccine (3 × 10^5^ pfu (low dose of vaccine; *n* = 51), 10^7^ pfu (high dose of vaccine; *n* = 35), 5 × 10^7^ pfu (high dose of vaccine, *n* = 16) or placebo (saline, *n* = 13)). In this study we used samples drawn from both high-dose groups. The study was approved and overseen by the Ethics Commission of the Canton of Geneva, Switzerland (14-221); World Health Organization’s Ethics Review Committee (RPC-696), and complied with all relevant regulations for work with human participants. Written informed consent was obtained from all participants.

### 2.2. RNA Extraction, Library Preparation and Sequencing

Whole blood samples were collected from the 51 volunteers in the high dose group, vaccinated with either 10^7^ or 5 × 10^7^ PFU of rVSVΔG-ZEBOV-GP, and from the 13 placebo. Blood samples were taken at day 0, day 1, day 3, day 7, day 14, day 28, day 35, and day 168 post immunization and stored at −80 °C in PAXgene Blood RNA kit (PreAnalytiX, QIAGEN, Hombrechtikon, Switzerland). Total RNA extraction was performed using PAXgene blood RNA Kit (PreAnalytiX, QIAGEN), according to manufacturer’s protocol (PAXgene Blood RNA kit Handbook, version 2, 2015). After extraction, RNA was quantified using the Qubit^®^ 2.0 Fluorometer with the RNA HS Assay Kit (Thermo Fisher Scientific, Waltham, MA, US).

Moreover, 50 ng of total RNA was reverse transcribed to produce cDNA, using SuperScript™ VILO™ cDNA Synthesis Kit (Thermo Fisher Scientific). cDNA was used to prepare the libraries with the Ion AmpliSeq™ Transcriptome Human Gene Expression Kit (Thermo Fisher Scientific), according to manufacturer’s recommendation, this kit allows the amplification of 20,812 human genes following 10 cycles of PCR amplification. Libraries were barcoded with Ion Xpress™ Barcode Adapters in order to sequence eight samples on each chip, the barcoded libraries were purified using Agencourt AMPure XP Magnetic Beads (Beckman Coulter, Brea, CA, US). The quantity of each library was evaluated using Ion Library TaqMan Quantitation Kit (Thermo Fisher Scientific) on a ViiA 7 Real-Time PCR System (Thermo Fisher Scientific).

Libraries were diluted to a concentration of 40 pM and pooled in groups of eight for sequencing on chips (Ion PI Chip Kit v3, Thermo Fisher Scientific), which were loaded using Ion Chef System (Thermo Fisher Scientific) and the IonPI Hi-Q Chef Kit (Thermo Fisher Scientific) according to manufacturer’s protocol. Sequencing reactions were performed on an Ion Proton Sequencer (Thermo Fisher Scientific) using Ion PI Hi-Q Sequencing 200 Kit (Thermo Fisher Scientific).

### 2.3. Data Exploration and Differential Gene Expression Analysis

Raw data counts, obtained from Ion Proton Sequencer, were organized in a counts table (File S1, available at [33]) containing gene names in the rows and different samples in the columns, where “counts” represent the total number of reads aligning to each gene. Sample information (i.e., sample name, study day, treatment, gender, sequencing batch, age) were reported in a descriptive table (File S2, available at [33]). Transcriptomic analysis was performed on samples from day 0 to day 28 after vaccination, including placebo and subjects vaccinated with either 10^7^ or 5 × 10^7^ pfu of rVSVΔG-ZEBOV-GP. The statistical analyses were performed in the R software environment (version 3.5.1). Differential expression analyses (DEAs, File S1–S3) were performed with edgeR (version 3.24.1), a package implemented in R [34,35]. Count data were normalized using the default edgeR method, named trimmed mean of M values (TMM), which estimates scale factors between samples [36]. Genes that had >1 count per million in at least ten samples were retained for further analysis. This filtered table was used to explore data in order to detect outliers and relationships among samples using principal component analysis (PCA) or Multi-Dimensional Scaling (MDS) analysis with base R and edgeR, respectively. After normalization, dispersion and Biological Coefficient of Variation (BCV) were estimated including an experimental design matrix. This matrix explains experimental conditions as a set of variables necessary to perform the quasi-likelihood test to identify the DE genes [37], which provides a better type I error control. Sequencing batches and analysed timepoints were used as variables for the design matrix. The generalized linear model, implemented with the glmQLfit function, was used to perform differential expression comparison between groups. Since there was no difference at baseline between placebo and vaccines (File S2), differentially expressed genes were assessed for each time point against the pre-vaccination baseline using the glmTreat function with a fold-change threshold of 1.2 and were considered significant when the Benjamini–Hochberg corrected *p*-value was <0.05 and the absolute fold change value higher than log2 (1.2).

### 2.4. Gene Set Enrichment Analysis

The result tables obtained from DEA were used to perform the enrichment analysis (File S4) using tmod R package [38]. The package tests the significance of the enrichment of features called blood transcriptional modules (BTM) that are sets of coexpressed genes in a specific condition or in the same functional pathway. Genes were ranked by false discovery rate (FDR) and the Coincident Extreme Ranks in Numerical Observations (CERNO) test, a modification of the Fisher’s method, was applied, obtaining the significance of enrichment and the area under the curve of the genes in each module. The 346 BTM identified by Li and colleagues in a systems biology study on five human vaccines (yellow fever vaccine, two influenza vaccines, and two meningococcal vaccines) were evaluated [26]. In each enriched BTM the number of up- or down-regulated genes was calculated based on the value of log fold change and FDR obtained from DEA.

### 2.5. Correlation Analysis

Correlation BTMs and antibody titers were assessed with the nonparametric Spearman’s Rho coefficient. Antibody titers were measured as described in [14] with an ELISA for EBOV-glycoprotein-specific antibodies using the homologous Zaire–Kikwit strain glycoprotein following the U.S. Army Medical Research Institute for Infectious Diseases’ (USAMRIID) standard operating procedure (SOP AP-03-35-00; USAMRIID ELISA). For each volunteer, a module activity score was calculated to measure BTMs activity and defined as increase or decrease of gene expression compared to baseline level (Day 0). To calculate module activity scores, genes with low expression levels (<1 count per million (CPM) in at least 50 samples) were filtered out. Gene expression was then transformed in log2 CPM and subsequently normalized in Z-scores (i.e., across all samples, each gene has a mean of 0 and a standard deviation of 1).

A mean of the Z-scores of each gene within a module was calculated obtaining a score of modules for each samples. Finally, a module activity score at Day X was defined for each module in each volunteer as scoreDX-scoreD0. Spearman correlation was performed between module activity scores and the fold increase at day 28 of USAMRIID GP-ELISA titers (compared to the baseline value) with the corr.test function of the R package psych. The obtained *p*-values were adjusted for multiple test with the Benjamini–Hochberg method.

### 2.6. Data and Materials Availability

All data associated with this study are available in the main text or the Supplementary Materials. Transcriptomic data are available in the Zenodo database at https://doi.org/10.5281/zenodo.3974486.

## 3. Results

### 3.1. Unsupervised Analysis Identified Day 1 Post-Vaccination as the Time Point with Highest Transcriptomic Changes

**Figure 1:**
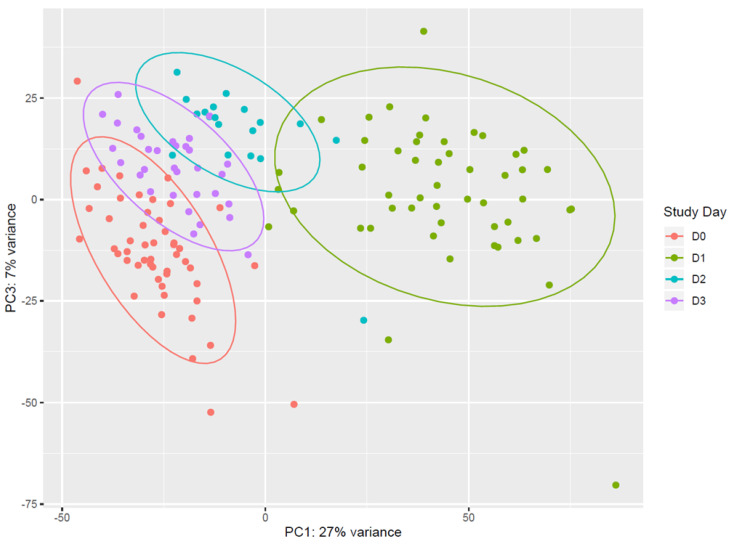
Principal component analysis of gene expression. Data from pre-vaccination (D0, red) and from the first 3 days after vaccination (D1, green; D2, light blue; D3, purple.) are reported in the principal component analysis (PCA) plot. Each dot represents a sample and the distance between samples reflects the variance in gene expression. Plot indicates a clear clustering of data by day of sampling. D0 and D1 data are very well separated along the PC1 axis, and appear more distributed along the PC3 axis than D2 and D3. Data from D2 and D3 are well separated from D1, while they tend to cluster with D0. Coloured ellipses define the normal confidence interval (95%) for each group of samples. Principal Component 1 (27% of total variance) and Principal Component 3 (7% of total variance) were chosen for this plot, as the plot produced using Principal Component 1 and Principal Component 2 (11% of total variance) was less discriminative (data not shown).

Vaccination with rVSVΔG-ZEBOV-GP induces strong transcriptomic changes in the blood of vaccinees. Unsupervised analysis using all expressed genes revealed that days 1, 2, and 3 after vaccination (D1, D2, D3) clustered separately from each other and from pre-vaccination samples (D0). Principal component analysis (PCA) showed that (i) D0 and D1 data were clearly separated, and that (ii) D2 and D3 data were separated from D1, while they tended to cluster with D0 (Figure 1). On the other hand, data from samples obtained at day 7, day 14, day 21, and day 28 all clustered together and with day 0 (Figure S1). The overall pattern of major gene expression shifts occurring after vaccination obtained by PCA indicated that upon vaccination there is an immediate change in gene expression which is evident at day 1, lasts until day 3, and essentially normalizes back to pre-vaccination conditions by day 7.

### 3.2. Vaccination with rVSVΔG-ZEBOV-GP Induces a Persistent Blood Transcriptomic Signal

**Figure 2:**
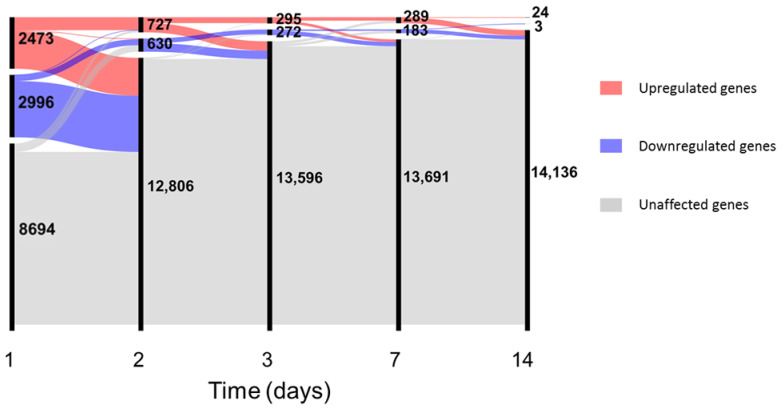
Number of differentially expressed genes up to day 14. In the alluvial plot, each vertical line represents a time point, the line is broken in sections proportional to the number of upregulated (red), downregulated (blue) and unaffected (grey) genes. Changes in composition of the different sections are shown by the lines connecting the time points. Data for plot construction were retrieved from the glmTreat analysis with edgeR. Days 21 and 28 are not shown since no differentially expressed genes (DEGs) were detected.

In-depth investigation of differential gene expression was performed using edgeR [34] with generalized linear models after filtering the unexpressed genes (see Supplementary Files and data for complete results). Since no major differences in gene expression were observed between genders at baseline (see File S1), samples from male and female volunteers were analysed together. At day 1, vaccination with rVSVΔG-ZEBOV-GP induced differential expression of 5469 genes, which constitute 38.6% of the total number of expressed genes; the number of differentially expressed genes decreased steadily over time from vaccination to 1357 at day 2, 567 at day 3, 472 at day 7, 27 at day 14, and no differentially expressed genes (DEGs) were detected after this time point (Figure 2).

Twenty-two genes were differentially expressed at all the time points from day 1 to day 14; Table 1 reports the gene names, their annotation and the values of log2 fold-change for each time point. Fifteen out of these 22 genes are involved in the interferon antiviral response, while the remaining 7 genes code for a monocyte chemotactic factor (CCL2), for a marker of monocyte activation (SIGLEC1), for factors involved in differentiation of monocytes (CMPK2), in polarization of macrophages to M1 phenotype (EPSTI1), in T-cell development (LY6E), in TNF-α dependent apoptosis (XAF1), and in the development of cellular junctions (AGRN). Out of 5469 differentially expressed genes at day 1, 4437 (81%) were uniquely found at this time point, while all the other time points had at least 78.4% of genes which were shared with one or more time points (Figure S2). The transcriptomic signal detected at the blood RNA level was highly intense at day 1 and returned to an expression similar to baseline only at day 21. These results suggest that immunization with the rVSVΔG-ZEBOV-GP vaccine strongly affects blood gene expression at 1 day after injection and continues to have detectable effects until two weeks. The massive transcriptomic response on the day following vaccination corresponds to the timing of occurrence of mild to moderate reactogenicity events (chills, fever, headache, fatigue or myalgia) in 50 out of 51 vaccinees [14]. Comparison of 5 × 10^7^ pfu dose versus 10^7^ pfu dose did not yield any differentially expressed gene, except for the MZB1 gene, coding for Marginal Zone B And B1 Cell Specific Protein, which was significantly higher (FDR = 0.04, log2 fold change = 1.8) in the 5 × 10^7^ pfu group at day 3 after vaccination.

### 3.3. Module Enrichment Analysis Reveals Long-Lasting Activation of Innate Immune Pathways

**Figure 3:**
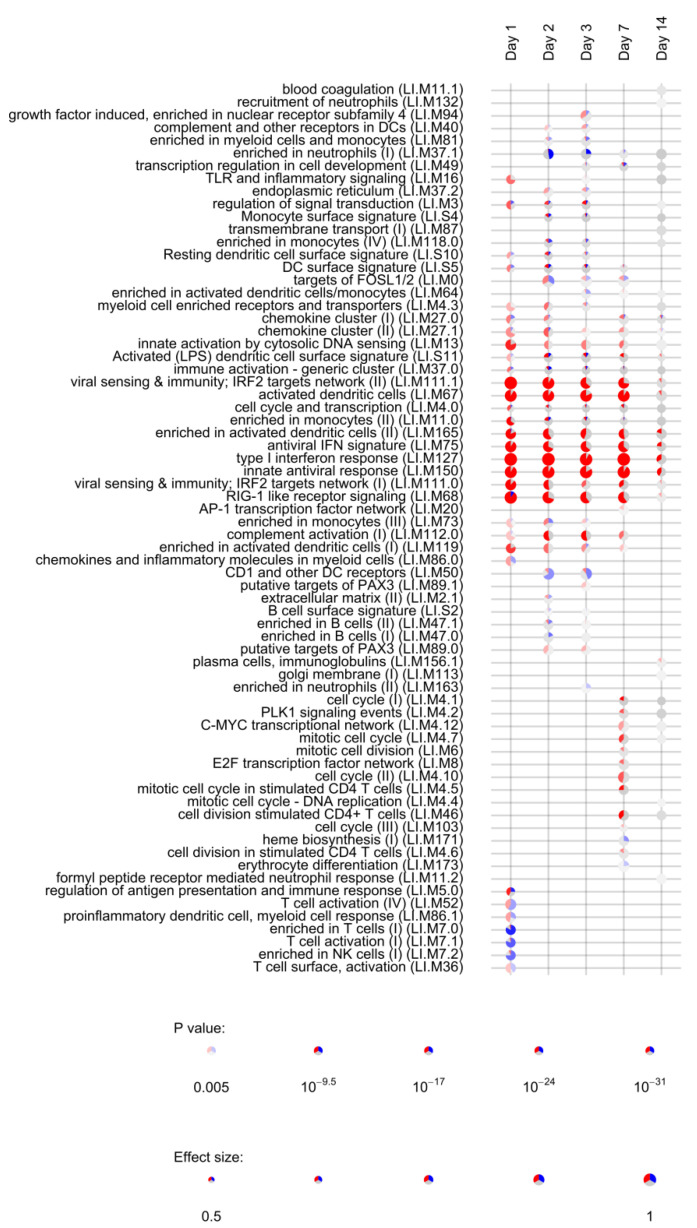
Activation of blood transcription modules by rVSVΔG-ZEBOV vaccination. Each column represents a study day after vaccination. Activation of modules was tested using the false discovery rate (FDR)-ranked lists of genes generated by edgeR glmTreat fitting and applying the CERNO test. Rows indicate different blood transcription modules, which were significantly (FDR < 0.005) activated in at least one time point. Each module is represented by a pie in which the proportion of significantly upregulated and downregulated genes is shown in red and blue, respectively. The grey portion of the pie represents genes that are not significantly differentially regulated. The significance of module activation is proportional to the intensity of the pie, while the effect size (Area Under the Curve) is proportional to its size.

Lists of differentially expressed genes obtained for each timepoint (day 1, day 2, day 3, day 7, day 14, day 21, and day 28) were used to assess the enrichment of blood transcription modules (BTMs) using the tmod R package [38]. BTMs include 346 sets of coordinately expressed genes which exert a specific function, described by the module title [26]. In total, 144 different BTMs were significantly (FDR < 0.05, File S4) enriched after vaccination with rVSVΔG-ZEBOV-GP. Of those, 22 remained activated up to day 14, while 21 were uniquely induced at day 1; 8 were uniquely enriched at day 2; 9 at day 3; 7 at day 7, and 8 at day 14. Interestingly, the number of DEGs was not correlated to the number of enriched BTMs, which was higher at day 3 after vaccination (File S5 and Figure S3). Figure 3 reports the most significantly enriched modules (FDR < 0.005) in at least one study time point. Modules associated to innate antiviral response, interferon response and dendritic cells were significantly enriched at day 1 through day 14, indicating a persistent activation of innate immunity. The retinoic acid-inducible gene I (RIG-I) like receptor signalling module (M68), which is considered to respond to viral nucleic acids, was also activated until day 14. This module is composed of ten genes, of which 9 were upregulated at day 1, while one (IL-8) was downregulated at day 1.

Persistent activation of modules until day 14 was mostly related to the above mentioned 22 genes (Table 1) which were differentially expressed at all time points. One module related to complement activation was also upregulated from day 1 to day 7. Five modules related to T cells and NK cells were largely downregulated at day 1, consistent with the lymphopenia observed at day 1 in rVSVΔG-ZEBOV-GP vaccinees [14]. At days 2 and 3 there was a significant downregulation of the “enriched in neutrophils” module, consistent with the neutropenia observed at day 3 after vaccination. At day 7, eight modules related to cell cycle and stimulation of CD4+ T cells were activated, compatible with the activation of adaptive immune responses. At day 14, one module related to plasma cells and immunoglobulins production was slightly upregulated possibly reflecting initiation of antibody production. When considering genes uniquely differentially expressed at a single time point, enrichment analysis identified only a single module (enriched in monocytes II, LI.M11) activated at day 1, suggesting that the most relevant genes for the activation of immune pathways are differentially expressed in at least two time points.

### 3.4. B Cell Activation and BCR Signalling Modules Correlate with Anti-EBOV-GP Antibody Titers

**Figure 4:**
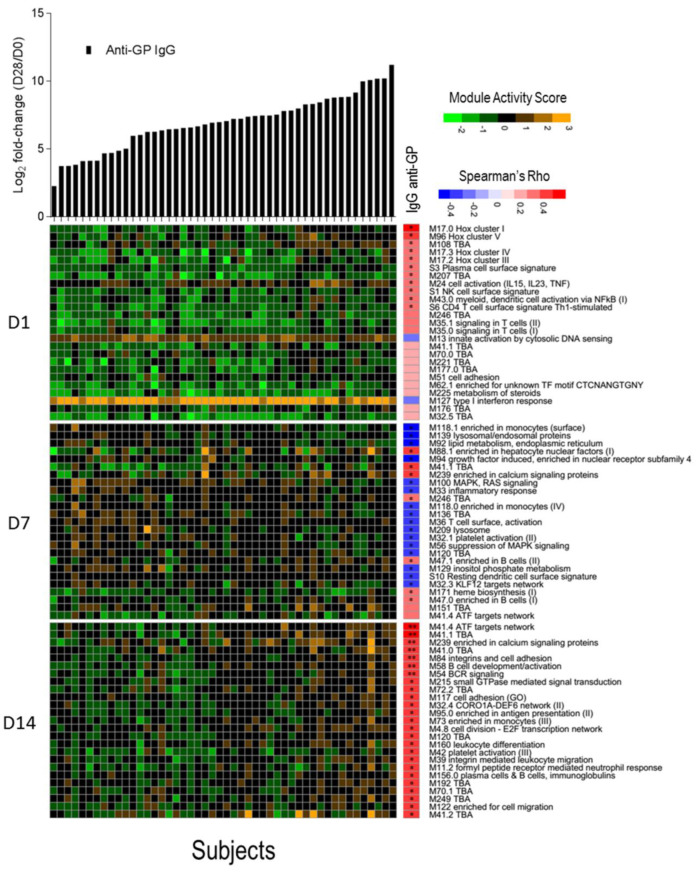
Correlation between activation of blood transcription modules (BTMs) and antibody titres. The gene expression of gene members of each module was collapsed in a single module activity score, defined as the mean value of normalized gene expression of gene member compared to the baseline level. Positive score indicates an increase in gene expression, negative score a decrease. Heat map of module activity scores (rows) and subjects (columns). Upper panel shows log2 fold increase (day 28/day 0) in each subject of total IgG anti-glycoprotein (GP) (in black). Subjects are ranked by fold increase of the total IgG anti-GP. A correlation analysis was performed calculating Spearman’s Rho coefficient between fold increase in antibody titres and module activity score. For each day, the top 25 modules ranked by absolute Spearman’s Rho correlation with anti-GP IgGs (right panel) are reported. Statistical significance of each correlation is reported within the correlation boxes ** = adjusted *p*-value < 0.05 (Benjamini–Hochberg method), * = *p*-value < 0.05.

We investigated whether a transcriptomic signal could be correlated with the magnitude of total anti-ZEBOV GP IgGs after vaccination using the blood transcription modules framework. For each subject, a module activation score was calculated at days 1, 7, and 14 and correlated with the fold-change in anti-ZEBOV GP IgGs. No correlation was found with antibody titers at 6 months and 1 year after vaccination, while correlations were identified with antibodies measured at day 28. After correction for multiple testing, seven significantly directly correlated modules were identified at day 14, including two modules related to B cell activation and BCR signalling, one related to activating transcription factor (ATF) target network, one enriched in calcium signalling, one in cell adhesion and two without annotation (Figure 4). Correlations were identified also with module activation scores at days 1 (10 modules) and 7 (23 modules), but these were no longer significant after multiple testing error correction. At day 1, although not significant, the strongest correlated modules were related to the Class I homeobox (Hox) signalling pathway, which is a family of transcriptional regulators involved in cell development and in the maturation of lymphoid cells, in particular of T cells [39]. At day 7, most modules were inversely correlated with antibodies and were related to monocytes, lysosomes, and mitogen-activated protein kinase (MAPK) signalling. The above significant direct correlation of anti-ZEBOV GP IgGs with B cell activation and BCR signalling in the day 14 data set could be expected since antibody production is dependent on the activation of B lymphocytes. Other modules that were significantly correlated at day 14 involve pathways such as calcium signalling, cell adhesion and activating transcription factor networks, which are possibly related to signal transduction.

## 4. Discussion

In this work, using a global transcriptomic approach, we profiled the host response to high dose vaccination (10^7^ and 5 × 10^7^ pfu) with rVSVΔG-ZEBOV-GP in volunteers of the phase 1/2 randomised controlled trial (NCT02287480) conducted in Geneva. We showed that rVSVΔG-ZEBOV-GP vaccination: (i) had a tremendous impact on the blood transcription in vaccinees, hijacking the host transcriptional homeostasis; (ii) had a sustained impact on innate immunity associated gene modules which possibly resulted in increased protection from viral infection; and (iii) induced a transcriptional signature of adaptive immune response that correlated with anti-GP antibody titers.

Indeed, vaccination with rVSVΔG-ZEBOV-GP causes a pauci-symptomatic viral infection, which was associated with a highly differential and consistent blood gene expression patterns in all vaccinees. Comparison of gene expression levels showed that vaccination produces a peak of 5469 DEGs at day one, representing 38.6% of the genes expressed by the host. The number of DEGs dropped steadily thereafter, with values of 1357 at day 2, 567 at day 3, 472 at day 7, 27 at day 14, and 0 at day 28. Over 40% of BTMs (144 out of 346) were significantly affected by vaccination. Gene expression was affected up to at least two weeks after immunization and pathway analysis showed that innate immunity and antiviral responses were the most prominent components involved in the immune response to rVSVΔG-ZEBOV-GP. Twenty-two genes were upregulated at all the time points from day 1 to day 14 (Table 1), these are mostly (15 out of 22) related to the interferon antiviral response, but are also involved in monocyte chemotaxis (CCL2), differentiation (CMPK2) and activation (SIGLEC1), polarization of macrophages to M1 phenotype (EPSTI1), T-cell development (LY6E), TNF-α dependent apoptosis (XAF1) and development of cellular junctions (AGRN). SIGLEC1 (CD169) is specifically expressed by a subpopulation of activated macrophages, and its surface expression has been shown to be significantly correlated with the vaccine dose [14]. Recently, SIGLEC1 was shown to interact with sialylated gangliosides on the Ebolavirus envelope and to control the uptake and entry into activated dendritic cells [40]. CXCL10 and CCL2 genes, whose gene products are involved in chemoattraction of monocytes and stimulation of inflammation, were strongly upregulated after vaccination. While the wild type VSV has a broad cellular tropism, it may be possible that ZEBOV glycoprotein expressed on the surface of rVSVΔG-ZEBOV-GP increases tropism of the recombinant virus for monocytes, thus inducing the observed high expression of monocyte-related genes and the activation of monocyte/dendritic cells related modules. Since VSV is used as a vector for other vaccines [41], it could be interesting to compare and validate the findings of the present study to other vaccine trials where VSV expresses a different heterologous antigen on its surface.

Among innate immune pathways, we showed that RIG-I was affected by rVSVΔG-ZEBOV-GP until day 14 after vaccination. Interestingly, the gene coding for IL-8, which is part of the inflammatory response of the cells after activation of RIG-I, was significantly downregulated at day 1 and not differentially expressed at the other time points. On the other hand, CXCL10, and TNF-α, which are also proinflammatory cytokine genes in the RIG-I pathway, were upregulated after vaccination. High levels of IL-8 were previously associated with poor prognosis after Ebola virus infection in humans [42,43]. It can be hypothesized that IL-8 is an essential proinflammatory factor in shifting the immune response towards the cytokine storm which is characteristic of Ebola virus disease.

Another manuscript focusing on a selected panel of 144 genes involved in vaccine responses, using a quantitative dual-color Reverse Transcriptase Multiplex Ligation-dependent Probe Amplification (dcRT-MLPA) platform, analyses the response to different vaccine doses of rVSVΔG-ZEBOV-GP in European, African, and North-American vaccinees cohorts and further confirms the importance of innate immune response for this vaccine, cross-validating the high gene expression level of innate response related genes (Vianello et al., submitted).

A recent transcriptomic study on 20 volunteers immunized with rVSVΔG-ZEBOV-GP identified the expression level of the TIFA gene (TRAF Interacting Protein With Forkhead Associated Domain, an adapter protein involved in both innate and adaptive immunity) at day 1 after vaccination as strongly correlated to antibody titres after 1 month [28]. We found that TIFA was significantly upregulated at day 1, but its expression level did not correlate with antibody titres at any of the analysed time points.

Modules related to innate antiviral response, to interferon response and to monocytes and dendritic cells were significantly enriched for the two weeks following vaccination, reflecting a long-lasting activation of innate immunity. Activation of innate immunity was also observed with the live attenuated Yellow Fever vaccine, for which differential gene expression peaked at day 7 with 65 genes shared at all the analysed time points [44], of those, 50 were also activated by rVSVΔG-ZEBOV-GP (Table S1). A complement activation module was also upregulated from day 1 to day 7. The most significantly down-regulated modules at day 1 were related to T cells and NK cells. This finding is in line the reported lymphopenia at day 1 after vaccination [14] and with the flow cytometric data which showed that rVSVΔG-ZEBOV-GP elicits a rapid and dose-dependent NK cell modulation [25]. At days 2 and 3, neutrophil modules were downregulated and complement-related modules upregulated. T-cell and cell-cycle associated modules were upregulated at days 7 and 14, while at day 28 no modules remained activated.

Correlates of protection of rVSVΔG-ZEBOV-GP are yet unknown, it is however likely that anti-ZEBOV GP IgGs are a major factor contributing to protection from disease. Seroconversion was detected for all of the vaccinees by day 28 after vaccination and specific IgGs could be detected up to two years after vaccination [14,20]. We identified seven modules whose activation at day 14 was directly correlated with anti-GP IgGs detected at 28 after immunization correlation analysis identified seven blood transcription modules, including two related to B cell activation and BCR signalling, at day 14 after vaccination which were directly correlated with ZEBOV glycoprotein-specific antibody titres. Two modules were related to B cells, two were related to signal transduction, one to cell adhesion and two have not been yet annotated and characterized. No significantly correlated modules were identified at earlier time points, possibly suggesting that the magnitude of transcriptional activation of innate immunity modules is not proportional to the subsequent adaptive response. Transcriptomic signatures identified in the present study may be shared with other vaccines and recapitulate relatively unspecific responses to vaccination, such as innate immune response and activation of B cells.

## 5. Conclusions

This is the first study profiling the blood transcriptomic response up to one month after rVSVΔG-ZEBOV-GP vaccination, providing an important contribution to the identification of immune signatures of the rVSVΔG-ZEBOV-GP that is the only Ebola vaccine approved for clinical use. This information could also have broader implications to support the identification of immune signatures of other Ebola vaccines.

## Figures and Tables

**Table 1:** Log_2_ fold-change values of the 22 genes differentially expressed from day 1 through day 14.

Gene	Annotation	Log2 Fold-Change				
		Day 1	Day 2	Day 3	Day 7	Day 14
AGRN	Development of neuromuscular junction	4.42	2.7	1.84	2.43	1.45
CCL2	Chemotactic factor for monocytes and basophils	7.91	5.13	2.79	3.42	2.10
CMPK2	nucleotide synthesis salvage pathway gene/role in terminal differentiation of monocytic cells	5.60	4.16	3.17	3.36	1.99
EPSTI1	M1 macrophage polarization gene	4.18	3.15	2.46	2.42	1.33
HERC5	IFN-induced, positive regulator of innate antiviral response	5.86	3.37	2.29	2.93	1.37
IFI27	Involved in type-I interferon-induced apoptosis	4.57	5.74	7.10	5.80	5.49
IFI44	IFN-induced, forms microtubular structures	4.76	4.23	3.60	3.41	1.97
IFI44L	IFN-induced, antiviral activity	5.53	5.04	4.29	4.06	2.42
IFI6	IFN-induced, role in apoptosis, antiviral activity	5.28	3.62	2.77	2.76	1.53
IFIT1	IFN-induced, antiviral RNA-binding protein	6.12	4.11	3.12	3.41	1.84
IFIT3	IFN-induced, antiviral and antiproliferative protein	5.12	3.43	2.54	2.7	1.50
ISG15	IFN-induced, ubiquitin-like protein, antiviral activity, induces NK cell proliferation, chemotactic factor for neutrophils and IFN-gamma-inducing cytokine	6.67	4.35	3.77	3.64	1.93
LY6E	Involved in T-cell development	3.95	3.53	3.09	3.03	1.87
MX1	IFN-induced, dynamin-like GTPase with antiviral activity	5.34	3.36	2.51	2.89	1.64
OAS1	IFN-induced, antiviral enzyme, regulator of apoptosis, cell growth, and differentiation	4.43	3.38	2.60	2.71	1.37
OAS2	IFN-induced, antiviral enzyme, inhibitor of protein synthesis	4.49	3.44	2.52	2.53	1.47
OAS3	IFN-induced, antiviral enzyme	5.48	3.83	2.93	3.27	1.93
OASL	IFN-induced, antiviral activity	5.33	3.18	2.33	2.52	1.12
RSAD2	IFN-induced, iron-sulphur (4FE-4S) cluster-binding antiviral protein, promotes production of IFN-beta production in plasmacytoid dendritic cells (pDCs), plays a role in CD4+ T-cells activation and differentiation	6.78	4.87	3.80	4.27	2.53
SIGLEC1 (CD169)	Mediates clathrin dependent endocytosis and sialic-acid dependent binding to lymphocytes	5.92	5.04	4.37	4.02	2.61
USP18	Regulation of inflammatory response to type 1 IFN	6.71	4.43	3.20	3.54	1.89
XAF1	Mediates TNF-alpha-induced apoptosis	3.88	3.17	2.59	2.46	1.20

## Data Availability

The data presented in this study are openly available in Zenodo at [doi:10.5281/zenodo.3974487], reference number [33]. Code used for data analysis is available in supplementary Files S1–S5.

## Supplementary Materials

The following are available online at https://www.mdpi.com/2076-393X/9/2/67/s1, Figure S1: Principal component analysis of gene expression data from days 0, 7, 14, 21, and 28, Figure S2: Number of differentially expressed genes shared among the different study days, Figure S3: Enrichment of BTMs by study day. Table S1: shared differentially expressed genes between YF17D and rVSVΔG-ZEBOV-GP, File S1: differential gene expression analysis of male versus female volunteers at day 0, File S2: DEA between placebo and vaccinated samples at day 0, File S3: Differential gene expression analysis of rVSVΔG-ZEBOV-GP vaccinated volunteers, File S4: enrichment analysis of differentially expressed genes in vaccinated subjects, File S5: enriched blood transcription modules at each study day.
