# Supplementary material for: Human Transcriptomic Response to the VSV-Vectored Ebola Vaccine
Source: Vaccines (Basel). 2021 Jan 20;9(2):67. doi: 10.3390/vaccines9020067 (PMC7908976; doi:10.3390/vaccines9020067)
Supplement: Supplementary file 1 [file vaccines-09-00067-s001.zip › Figure S3.pdf]

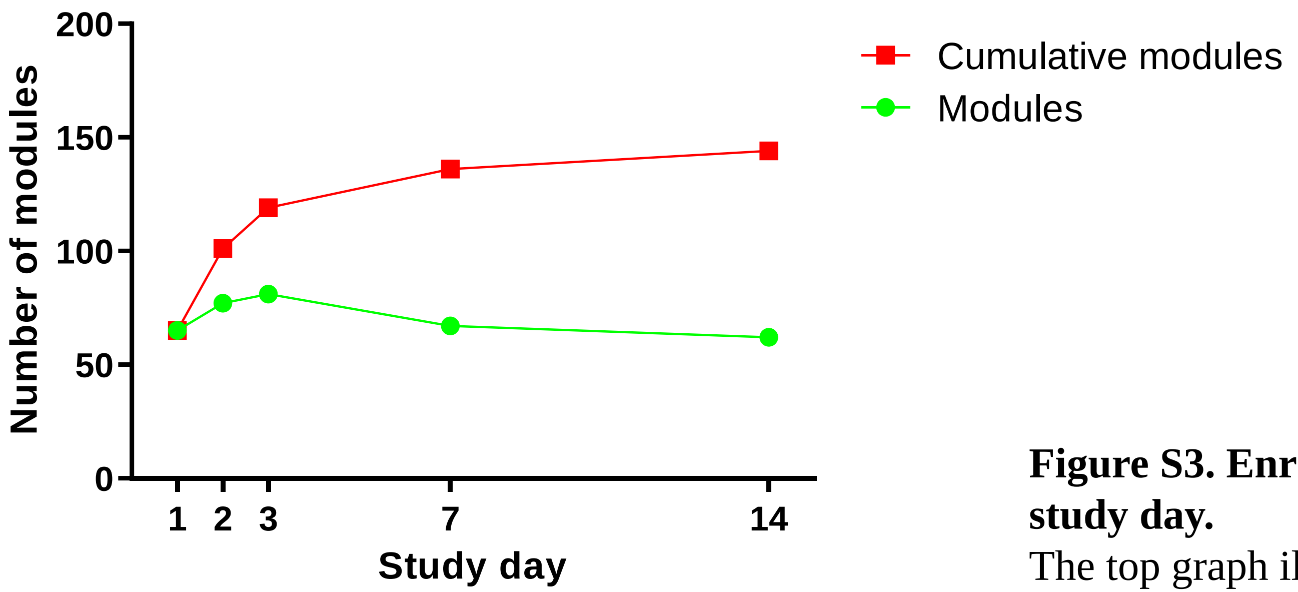

Day	Significant Modules	Cumulative modules
1	65	65
2	77	101
3	81	119
7	67	136
14	62	144

Figure S3. Enrichment of BTMs by study day.

The top graph illustrates the number of significantly (FDR < 0.05) enriched modules at each analyzed time point (green line). Overall 144 different modules become activated in the two weeks following vaccination. The red line represents the number of unique BTMs that become enriched, reflecting the evolution of the immune response over time.

Number of enriched BTMs are reported in the table below.
